# A Lebanese dietary pattern promotes better diet quality among older adults: findings from a national cross-sectional study

**DOI:** 10.1186/s12877-016-0258-6

**Published:** 2016-04-19

**Authors:** Lamis Jomaa, Nahla Hwalla, Leila Itani, Marie Claire Chamieh, Abla Mehio-Sibai, Farah Naja

**Affiliations:** Department of Nutrition and Food Sciences, Faculty of Agricultural and Food Sciences, American University of Beirut, P.O. Box 11–0.236, Riad El Solh 11072020, Beirut, Lebanon; Department of Nutrition and Dietetics, Faculty of Health Sciences, Beirut Arab University, Beirut, Lebanon; Department of Epidemiology and Public Health, Faculty of Health Sciences, American University of Beirut, P.O. Box 11–0.236, Riad El Solh 11072020, Beirut, Lebanon

**Keywords:** Dietary quality, Dietary patterns, Older adults, Lebanon

## Abstract

**Background:**

Proper nutrition is critical for healthy aging and maintaining functional independence. Limited research has been done on the assessment of dietary patterns of older adults and their association with diet quality indices. This study was conducted to identify, characterize, and evaluate the dietary patterns and diet quality of older adults in Lebanon, a middle-income country undergoing nutrition transition.

**Methods:**

A cross-sectional population-based study was conducted on a nationally representative sample of community-dwelling older adults aged >50 years (*n* = 525). Socio-demographic, anthropometric, and lifestyle variables were collected through interviews, and dietary intake was assessed using a semi-quantitative food frequency questionnaire (FFQ). Five commonly used diet quality indices (DQIs) were calculated, including the Alternative Healthy Eating Index (AHEI), the alternate Mediterranean diet score (aMed), the Dietary Approach to Stop Hypertension (DASH) style diet score, and the Lebanese Mediterranean Diet index. Dietary patterns (DPs) were derived using exploratory factor analysis. Associations of identified DPs with energy, energy-adjusted nutrients, and DQIs were evaluated by Pearson’s correlations. Multiple linear regression analyses were used to examine correlates of DPs.

**Results:**

Three DPs were derived: Lebanese, Western, and High Protein/Alcohol patterns. The Lebanese pattern had highest correlations with fiber, folate, vitamin C, and all five DQIs. The Western was positively associated with energy and sodium and was inversely correlated with fiber, most vitamins, and a number of DQIs, namely AHEI, aMED, and DASH-style diet score. Highest correlations with intakes of proteins and fat were observed for the High Protein/Alcohol pattern. The Lebanese pattern was associated with female gender, education, nonsmoking and physical activity, whereas the Western pattern was associated with adverse health behaviors, including smoking, skipping breakfast, and physical inactivity.

**Conclusions:**

Of the three identified patterns, the Lebanese DP was associated with better diet quality and healthier lifestyle behaviors while the Western pattern implicated a lower quality diet. Public health programs promoting prudent diets, including the Mediterranean and Lebanese DPs, are needed to improve the diet quality of middle-aged and older adults in an attempt to improve their functionality and quality of life.

## Background

Older adults, aged 60 years and over, represent the fastest growing segment of the global population with numbers projected to reach 1.5 billion by year 2050 [1]. This unprecedented trend of population aging in today’s world necessitates a reduction in the burden of diseases and disabilities to ensure longer and healthier lives [1, 2]. Healthy or ‘successful’ aging represents a state of resilience whereby cognitive and physical functioning is maintained and the risk of chronic diseases is reduced [3]. Proper nutrition is one of the main behavioral and modifiable determinants of healthy aging and is integral in reducing disease risk and maintaining functional independence, especially among older adults who undergo a variety of physiological, psychological, and social changes that affect their nutritional status. These changes include alterations in appetite hormone levels, reduced gastrointestinal digestive and absorptive capacities, increased feelings of loneliness and depression among other complex biological, metabolic, and environmental factors [1, 4]. Numerous cross-sectional and cohort studies elucidate the strong association between adequate nutrition and reduced risk of diet-related chronic diseases [5, 6] with more emphasis being placed over the last few years on improvements in health-related quality of life parameters through diets of good quality [7–10].

Traditionally, diet quality has been assessed by comparing dietary intake of single nutrients or specific foods to dietary references. However, given that individuals consume foods, and foods are a combination of nutrients that may interact closely to affect health outcomes, recently nutrition epidemiologists have been assessing dietary quality through dietary patterns (DPs) looking beyond the intake of single nutrients or food items to characterize whole diets and habitual food consumption. Two approaches commonly used to assess dietary patterns are the *a priori-* and *a posteriori-* methods. The *a priori*-defined dietary patterns are based on compliance to pre-set international or national dietary recommendations and guidelines that have been associated with positive health outcomes [11]. Using this method, the diet can be assessed based on frequency of consumption of certain food and/or nutrients resulting in a scoring system that is operationalized as a diet quality index (DQI) [11, 12]. Examples of commonly used DQIs are the Healthy Eating Index [13], the Alternative Health Eating Index (AHEI) [5], the Mediterranean Diet Score [14], and the Dietary Approaches to Stop Hypertension (DASH) [15]. The *a posteriori-*defined dietary patterns (DPs), on the other hand, are not pre-defined but rather based on empirical data of populations under study and are derived through sophisticated statistical techniques, such as factorial or cluster analyses [12, 16]. These empirical methods allow for identifying culture and context-specific DPs based on combination of food items as consumed by the population under study. Empirically-derived DPs, most commonly reported in the nutrition literature, are the ‘prudent’ DP, characterized by high intakes of fruits, vegetables, whole grains, fish, and poultry; and the ‘Western’ pattern that is characterized by high intakes of refined cereals, sweets and desserts, as well as red and processed meats [16].

Lebanon, a small Eastern Mediterranean country, is undergoing a similar demographic transition as that seen worldwide with projections showing that older adults are expected to constitute 10 % of the population by 2025 [17]. Concomitantly, the country is witnessing increased prevalence of obesity and diet-related chronic diseases among various population groups, including middle-aged and older adults [18–20]. Despite the population ageing phenomena observed in Lebanon and the important role that nutrition plays in the prevention and treatment of diet-related diseases, limited studies examined diet quality of community-dwelling older adults [21]. Non-institutionalized older adults in Lebanon are vulnerable to malnutrition and inadequate dietary intake due to their limited access to health care services and social welfare programs as well as inadequate living conditions; however this group still receives minimal research attention. The proposed study aims to (i) identify and characterize dietary patterns of Lebanese older adults, (ii) evaluate the identified DPs in relation to diet quality indices, and (iii) examine socio-demographic factors and lifestyle behaviors as correlates of the identified DPs.

## Methods

### Study design

The data used in this study were derived from the Nutrition and Non-Communicable Disease Risk Factor Survey conducted in Lebanon between years 2008 and 2009 on a nationally representative sample of 3656 individuals aged 6 years and above. This sample was drawn from randomly selected households, based on stratified cluster sampling: the strata were the Lebanese Governorates and the clusters were selected further at the level of districts covering urban and rural areas. Households constituted the primary sampling units in the different districts of Lebanon. One adult from each household was selected from the household roster. The distribution of the national survey sample by sex and 5-year age group was similar to that of the Lebanese population as estimated by the Central Administration for Statistics in Lebanon (2004). Out of 3178 eligible subjects approached, 2836 accepted to participate (response rate 89.2 %). For the purpose of this study, only adults above 50 years of age were included (*n* = 565).

During face-to-face interviews, study participants completed a brief socio-demographic and lifestyle questionnaire and a semi-quantitative Food Frequency Questionnaire (FFQ). Interviews were conducted by trained dietitians and took place at the participants’ homes. Further details about the survey and data collection procedures were described elsewhere [22]. Socio-demographic and lifestyle information collected through the questionnaires included age (years), sex, education (illiterate, less than high school, high school or diploma, university level), marital status (single including single, divorced, separated and widowed, and married including currently married or living with a partner), and crowding index. The latter is a composite variable composed of the number of household members as the numerator and the number of rooms used for sleeping as the denominator. Several epidemiological studies have correlated a high household crowding index with low socioeconomic status (SES) [23]. Smoking status was either labeled as smokers (current smokers) or non-smokers (current non-smokers and past smokers). Physical activity was assessed using the short version of the International Physical Activity Questionnaire (IPAQ). Three categories of physical activity were assigned based on METS-min per week (low: less than 600, moderate: at least 600, and high: at least 3000) [24]. In addition, participants answered questions related to the presence of chronic diseases and their family history of chronic diseases, including obesity, heart diseases, hypertension and diabetes (a positive family history was indicated if the mother, father or both were reported as having the chronic disease).

Anthropometric measurements were taken using standardized techniques and calibrated equipment. Subjects were weighed to the nearest 0.1 kg in light indoor clothing and with bare feet or stockings. Using a stadiometer, height was measured without shoes and recorded to the nearest 0.5 cm. Body mass index (BMI) was calculated as the ratio of weight (kilograms) to the square of height (meters). Waist circumference was measured using a plastic measuring tape, to the nearest 0.5 cm, at the midpoint between the bottom of the rib cage and above the top of the iliac crest during minimal respiration. Elevated waist circumference, reflecting central obesity, was defined according to the International Diabetes Federation (IDF) cut-off values (WC ≥ 94 cm for males and ≥ 80 cm for females) [25]. Percent body fat was computed using skinfold thickness measurements according to the Durnin and Womersley formula [26]. Skinfold measurements were performed on the right side of the body and at four sites (biceps, triceps, subscapular, and suprailiac) to the nearest 0.1 mm. The sex-specific cut-off points of 25 and 32 % body fat were used to indicate adiposity in men and women, respectively [25, 27].

Dietary intake of participants was assessed by a 61-item semi-quantitative, context-specific FFQ that has been used in previous studies among the Lebanese population and has yielded plausible results and findings [28, 29]. The FFQ referred to food intake one year prior to the interview, and for each food item listed, a standard portion size was indicated, and subjects were asked to record the frequency of consumption (open-ended) either per day, per week, per month, per year or never. Daily gram intakes of food items as well as the energy and nutrient composition of foods were computed using the food composition database of the Nutritionist IV software and the food composition table for local and traditional Middle-Eastern foods [30, 31]. FFQ used in the present study was administered by a trained dietitian and not self-completed. Such a modality of FFQ completion does not require a literate population, resulting in consistent interpretations, and higher response and completion rates, each of which may enhance the validity of the data. Breakfast frequency was also investigated in this study as it was shown to be associated with improved daily nutrition intake and reduced risks of obesity and type II diabetes among older adults [32].

### Dietary Quality Indices (DQIs)

Using dietary intake data derived from the FFQ, the following DQIs were selected: the Alternative Health Eating Index (AHEI), the alternate Mediterranean diet score (a-MED), the Dietary Diversity Score (DDS), the Dietary Approaches to Stop Hypertension (DASH)-style diet score, and the Lebanese Mediterranean Diet index (LMD). The selection of these scores was based on a thorough review of related literature to identify indices associated consistently with lower risk of chronic diseases in clinical and epidemiologic investigations. The scores of AHEI, aMED, DASH, DDS and LMD, were derived based on protocols defined earlier by Chiuve 2012 [5], Fung 2009 [14], Fung 2008 [15], Kant 1993 [33], and Naja 2014 [28], respectively. For all the aforementioned DQIs, a higher score represents a better dietary quality, and the ranges of scores and categories for each of the DQIs used in this study were presented in Table 1.

**Table 1:** Descriptive characteristics of a representative sample of Lebanese older adults (*n* = 525)^a^

	Total (*n* = 525)
*Demographic characteristics*	
Age (yrs) Mean ± SD	66.4 ± 7.89
50 + −64.9	263 (50.1)
≥ 65	262 (49.9)
Sex	
Male	268 (51)
Female	257 (49)
Education	
Illiterate	65 (12.4)
Less than high school	204 (38.9 %)
High school or Diploma	190 (36.2 %)
University level	66 (12.6)
Marital status	
Married	354 (67.4)
Single/Divorced/Separated/Widowed	171 (32.6)
Crowding index	
< 1 person/room	297 (57)
≥ 1 person/room	224 (43)
*Lifestyle characteristics*	
Physical activity	
Low intensity (less than 600 METs)	237 (45.5)
Moderate intensity (at least 600 METs)	191 (36.7)
High intensity (at least 3000 METs)	93 (17.9)
Smoking	
Never/Past smoker	319 (60.8)
Current smoker	206 (39.2)
Alcohol intake	
No alcohol	304 (57.9)
Occasionally	135 (25.7)
1–4 times per week	54 (10.3)
5 or more per week	32 (6.1)
*Presence of chronic disease* ^b^	422 (80.4)
Family history of chronic diseases^c^	
No	95 (18.1)
Yes	429 (81.7)
*Anthropometric measurements*	
Weight (kg)	76.42 ± 14.97
Height (cm)	161.61 ± 9.45
BMI (kg/m^2^) (Mean ± SD)	29.29 ± 5.48
BMI classification	
Underweight (<18.5 kg/m^2^)	8 (1.5)
Normal weight (18.5 ≤ BMI < 25 kg/m^2^)	91 (17.3)
Overweight (25 ≤ BMI < 30 kg/m^2^)	213 (40.6)
Obese (≥30 kg/m^2^)	213 (40.6)
Waist circumference (cm) ^d^ (Mean ± SD)	98.47 ± 13.21
Normal (Females < 80 cm, Males <94 cm)	112 (21.3)
Elevated (Females ≥ 80 cm, Males ≥94 cm)	413 (78.7)
Percentage body fat (%)^e^ (Mean ± SD)	36.02 ± 7.80
Normal (Females < 32 % & Males < 25 %)	53 (10.3)
High (Females ≥ 32 % & Males ≥ 25 %)	461 (89.7)
*Diet Quality Indices (DQIs)*	
Alternative healthy Eating Index (AHEI) (Score range:0–100)	52.61 ± 10.95
Low (≤50)	220 (41.9)
High (>50)	305 (58.1)
alternate Mediterranean Diet Score (aMED) (Score Range 0–9)	4.33 ± 1.76
Low (≤4.5)	266 (50.9)
High (>4.5)	257 (49.1)
DASH-style diet score (Score range: 8–40)	25.28 ± 4.29
Low (≤24)	237 (45.2)
High (>24)	287 (54.8)
Lebanese Mediterranean Diet Index (LMD) (Score Range 9–27)	17.72 ± 3.24
Low (≤18.0)	304 (57.9)
High (>18.0)	221 (42.1)
Dietary Diversity Score (DDS) (Score Range 0–5)	4.51 ± 0.75
Low (≤2.5)	13 (2.5)
High (>2.5)	512 (97.5)
Breakfast frequency per week ^f^ (Mean ± SD)	5.76 ± 2.39
Low (≤4 times/week)	116 (22.2)
High (>4 times/week)	407 (77.8)

^a^Categorical variables are presented as n (%) and continuous variables as mean ± SD

^b^Presence of chronic disease was defined as having at least one chronic disease, such as obesity, hypertension, diabetes, heart disease

^c^Family history of chronic diseases was defined as having a family member with at least one chronic disease, such as obesity, hypertension, diabetes, heart disease

^d^Elevated waist circumference was defined according to the IDF waist circumference threshold for obesity (Zimmet et al. [25])

^e^ Sex specific percentage body fat cut off points were used to identify subjects with high or normal body fat (Lohman et al. [27])

^**f**^ For the breakfast frequency measure, scores were grouped into two categories low and high corresponding to values below and above the median respectively

### Dietary patterns derivation

The exploratory Principal Component Factor Analysis (PCFA) was used to derive the DPs [16]. Food items were grouped into 26 food groups based on similarities in ingredients, nutrient profile and/or culinary usage (Appendix). Food items having a unique composition (eggs, burghol [parboiled wheat], and mayonnaise) were classified individually. The total consumption for each group was determined by summing up the daily portion intake of each item in this group. These food groups were entered into the PCFA. Prior to running the analysis, the correlation matrix between the 26 food groups was visually and statistically examined to justify undertaking factor analysis. The chi-square for Bartlett test of sphericity was significant at a *p*-value less than 0.05, and the Kaiser-Meyer-Olkin test (KMO) was greater than 0.6, indicating that the correlation among the variables was sufficiently strong for a factor analysis. The number of factors retained was based on three criteria: 1) the Kaiser criterion (eigenvalues >1), 2) inflection point of the scree plot 3) and the interpretability of factors. The factors were rotated by a Varimax rotation (orthogonal transformation). The derived DPs were labeled based on food groups having a rotated factor loading greater than 0.4 [34]. Factor scores were calculated using the multiple regression approach and each individual received a factor score for each DP. For each pattern, participants were grouped into quartiles of pattern scores.

### Statistical analysis

Continuous and categorical variables were described using means ± standard deviations (SD) and proportions, respectively. Pearson’s correlation coefficients were used to examine the association of the identified DP with energy, energy-adjusted nutrient intake, and the five DQIs. These correlation coefficients were compared using Steiger- based equations [35]. Multiple linear regression analyses were used to examine correlates of DPs adjusted for total daily energy intake. The dependent variables in these regression models were the factor scores of the identified patterns. Tests for linearity (Tolerance > 0.4) of the covariates included in the regression models were performed. Normality of the residuals was assessed by the histogram of standardized residuals and normal probability plot in all regression models. The Statistical Package for the Social Sciences (SPSS; version 14.1) was used for all computations and a *p*-value <0.05 was considered significant.

## Results

Out of a total of 565 survey participants aged 50 years and above, 525 (93 %) completed dietary intake data. Descriptive characteristics of study participants included socio-demographic and lifestyle characteristics, presence of chronic disease and family history of chronic diseases, as well as anthropometric measurements and DQIs, as presented in Table 1. Mean age of study participants was 66.4 ± 7.89 years with a comparable sex distribution (51 % males vs 49 % females). A higher percentage of study participants reported physical activity of low intensity compared to moderate and high intensity levels (45 % versus 36.7 and 17.9 %, respectively). Almost 40 % of study participants reported to be current smokers and alcohol consumers. A high percentage of participants in this study reported having at least one chronic disease (80.4 %) and 81.7 % reported having family history of chronic diseases including heart disease, diabetes, obesity, and hypertension. Furthermore, the proportion of study participants who were diagnosed as obese, based on a BMI greater than 30 kg/m^2^, was 40.6 % (*n* = 213), whereas a higher proportion of the study population (78.7 %, *n* = 413) had an elevated waist circumference, reflecting central obesity. Furthermore, the percentage of body fat was high among 89.7 % of the study population.

While the majority of study participants had a high dietary diversity score (97.5 %), approximately 50 % of the study population had low scores on the AHEI (41.9 %), aMED (50.9 %), DASH-style diet score (45.2 %) and the LMD (57.9 %). Using factor analysis, three DPs were identified. Factor loadings of the three patterns are shown in Table 2 (loadings greater than 0.3 are bolded). The classification and nomenclature of the patterns were based on food groups loading higher than 0.4 on the respective pattern [34]. Accordingly, the pattern characterized by a high intake of refined grains, fried potato, olives, sweets and soda beverages was named as Western DP. The pattern with high loadings of fruits, vegetables, starchy vegetables, dried fruits, bulgur, legumes, nuts and seeds was referred to as the Lebanese pattern. High protein/Alcohol nomenclature was given to the pattern that was positively associated with intakes of various kinds of meats, including red meat, poultry and fish, in addition to alcohol. The DPs identified explained together 25.55 % of the variance of dietary intake data in the study population (9.74, 8.03 and 7.78 % for the Western, Lebanese, High Protein/Alcohol patterns respectively).

**Table 2:** Factor loading matrix for the three identified dietary patterns in a representative sample of Lebanese older adults (*n* = 525)

	Dietary patterns		
	Western	Lebanese	High protein/Alcohol
Refined grains	**0.710**		
Bread whole	**−0.564**		0.213
Fried potato	**0.527**		0.256
Olives	**0.509**	0.272	
Sweets	**0.486**	0.237	
Soda regular	**0.435**		
Eggs	**0.354**		**0.311**
Pizza and pies	**0.329**		
Hot drinks	**0.327**		
Fast food	**0.318**	0.247	
Fat	**0.315**		0.230
Fruit juices bottled	0.227		
Breakfast cereals			
Fruit		**0.608**	
Vegetables		**0.565**	**0.328**
Starchy vegetables		**0.516**	
Dried fruits		**0.498**	
Bulgur		**0.381**	
Legumes		**0.380**	**0.365**
Nuts and seeds		**0.354**	**0.327**
Soda light		0.284	
Poultry			**0.646**
Meat			**0.613**
Fish			**0.484**
Alcohol			**0.456**
Dairy			0.277
Percent variance explained	9.74	8.03	7.78

Absolute values < 0.2 were excluded from the table for simplicity

Absolute values ≥ 0.3 are bolded

Pearson’s correlations for the association of the identified DPs with energy and energy-adjusted nutrients were compared using Steiger’s Z formula (Table 3). When compared to the Lebanese and the High Protein/Alcohol patterns, the scores of the Western pattern had the highest correlations with energy and sodium intakes and the lowest correlations with fiber, and vitamins A, D, K, C as well as all the B-vitamins, except for Vitamin B12 (see Table 4). Furthermore, the Western pattern had the lowest associations with calcium, iron, potassium, zinc, magnesium, manganese, and phosphorus. Among all the patterns, the scores of the Lebanese pattern had the highest correlations with fiber, folate, potassium, and vitamin C. The highest correlations with intakes of proteins, total fat, omega 3 fatty acids, and cholesterol were observed for the scores of the High Protein/Alcohol pattern. The latter pattern was also least associated with carbohydrate intake (Table 3).

**Table 3:** Associations between the identified dietary patterns with energy and energy-adjusted nutrient intakes and diet quality indices in a representative sample of Lebanese older adults (*n* = 525)

Nutrients	Western	Lebanese	High protein/Alcohol
Total energy (Kcal)	0.59^b,c^	0.44^b,d^	0.54^b,c^
Protein (g)	−0.29^b,c^	−0.07^d^	0.48^b,e^
Carbohydrates (g)	0.21^b,c^	0.20^b,c^	−0.43^b,c^
Fat (g)	−0.10^a,c^	0.03^d^	0.12^b,d^
Cholesterol (mg)	0.12^b,c^	−0.30^b,d^	0.31^b,e^
Saturated fat (g)	−0.01^c,d^	−0.09^a,c^	0.06^d^
Linoleic acid (g)	−0.09^a,c^	0.07^d^	0.05^d^
Linolenic acid (g)	−0.06	−0.03	0.06
Alcohol (g)	−0.17^b,c^	−0.23^b,c^	0.32^b,d^
Fiber (g)	−0.51^b,c^	0.57^b,d^	0.10^a,e^
Vitamin A (μg RE)	−0.14^b,c^	0.17^b,d^	0.19^b,d^
Vitamin D (μg)	−0.12^b,c^	0.09^a,d^	0.05^d^
Vitamin E (mg)	−0.07^c^	0.09^a,d^	0.02^d^
Vitamin K (μg)	−0.21^b,c^	0.38^b,d^	0.19^b,e^
Vitamin C (mg)	−0.20^b,c^	0.54^b,d^	−0.02^e^
Riboflavin (mg)	−0.21^b,c^	0.08^d^	0.16^b,d^
Niacin (mg)	−0.24^b,c^	0.07^d^	0.35^b,e^
Pyridoxine (mg)	−0.44^b,c^	0.50^b,d^	0.18^b,e^
Folate (μg)	−0.33^b,c^	0.49^b,d^	0.09^a,e^
Cobalamin (Vit B12) (μg)	−0.04^c^	−0.15^b,c^	0.31^b,d^
Sodium (mg)	0.19^b,c^	−0.16^b,d^	−0.9^b,e^
Calcium (mg)	−0.14^b,c^	0.11^a,d^	−0.01^e^
Iron (mg)	−0.22^b,c^	0.39^b,d^	0.32^b,d,e^
Zinc (mg)	−0.34^b,c^	0.03^d^	0.45^b,e^
Magnesium (mg)	−0.57^b,c^	0.49^b,d^	0.25^b,e^
Phosphorous (mg)	-.053^b,c^	0.26^b,d^	0.39^b,e^
Diet Quality Indices (DQIs)			
Alternative Healthy Eating Index (AHEI)	−0.50^b,c^	0.37^b,d^	−0.01^e^
Alternate Mediterranean Diet Score (aMED)	−0.09^a,c^	0.56^b,d^	0.21^b,e^
DASH-style Diet Score	−0.38^b,c^	0.46^b,d^	−0.07^e^
Lebanese Mediterranean Diet Index (LMD)	0.16^b,c^	0.67^b,d^	0.31^b,e^
Dietary Diversity Score (DDS)	0.02^c^	0.30^b,d^	0.29^b,d^
Breakfast frequency per week	−0.11^a,c^	0.041^d^	0.02^d^

^a^ Correlation is significant at the 0.05 level

^b^ Correlation is significant at the 0.01 level

^c, d, e^ Values with different superscripts are significantly different *p* < 0.05 (Using the Steiger’s Z formula to test the statistical significance of the difference between two dependent correlations)

**Table 4:** Correlates of the identified dietary patterns in the study population as assessed by multivariate linear regression (*n* = 525)

	Western		Lebanese		High protein/Alcohol	
Variable^a^	β	95 % CI	β	95 % CI	β	95 % CI
Age	−0.044	−0.190, 0.101	0.129	−0.039, 0.297	−0.081	−0.235, 0.073
Males versus females	0.271	0.112, 0.429	−0.353	−0.537, −0.169	−0.097	−0.266, 0.071
Education	−0.163	−0.252, −0.074	0.127	0.024, 0.230	0.103	0.009, 0.198
Marital status	0.031	−0.131, 0.194	−0.084	−0.272, 0.104	−0.065	−0.237, 0.107
Crowding index	0.177	0.037, 0.317	−0.098	−0.260, 0.064	−0.087	−0.236, 0.061
Frequency of breakfast consumption	−0.044	−0.073, −0.015	−0.005	−0.038, 0.029	−0.001	−0.032, 0.030
Physical activity level	−0.123	−0.213, −0.033	0.155	0.051, 0.259	−0.002	−0.097, 0.094
Smoking	0.240	0.095, 0.385	−0.187	−0.355, −0.019	0.037	−0.117, 0.191
Frequency of alcohol consumption	−0.174	−0.255, −0.093	−0.003	−0.097, 0.090	0.253	0.167, 0.339
Presence of chronic disease	−0.221	−0.394, −0.048	0.012	−0.189, 0.212	0.184	0.000, 0.368
Family history of chronic diseases	0.016	−0.160, 0.193	−0.015	−0.219, 0.189	0.065	−0.123, 0.252

^a^Values presented in this table are linear regression coefficients and their corresponding 95 % confidence intervals. All variables considered in these regression analyses are categorical except for age, frequency of breakfast consumption and the scores of the dietary patterns, which were continuous

Similar analyses were conducted for comparing the associations of the identified DPs with DQIs in the study population (Table 3). Compared to the Western and the High Protein/Alcohol patterns, the Lebanese pattern had the highest associations with scores of the AHEI (*r* = 0.366), aMED (*r* = 0.563), DASH (*r* = 0.458), and LMD (*r* = 0.669). On the other hand, the Western pattern had the lowest associations with the scores of the AHEI (*r* = −0.500), aMED (*r* = −0.094), DASH (*r* = −0.377), and LMD (*r* = 0.158) (Table 3).

The multivariate regression coefficients and their corresponding 95 % confidence intervals for the associations of the derived patterns with socio-demographic and lifestyle characteristics, as well as presence of a chronic disease and family history of chronic diseases are presented in Table 4. The scores of the Western pattern were positively associated with male sex, a higher crowding index, and smoking yet the scores of this pattern were negatively associated with education level, breakfast consumption, alcohol consumption, and presence of chronic diseases. As for the Lebanese pattern, higher scores were associated with education level and physical activity while male sex, crowding index, and smoking were negatively associated with the score of this pattern. Positive associations were observed between the scores of the High Protein/Alcohol pattern with education level and alcohol consumption. Using bivariate as well as multivariate logistic regression analyses, no association between any of the identified DPs and obesity indices (BMI, waist circumference and percent body fat) was observed (data not shown).

## Discussion

This study aimed to identify, characterize, and evaluate the DPs and diet quality of a representative sample of Lebanese older adults. Three DPs were identified in this study: a Western, a Lebanese, and a High Protein/Alcohol pattern. Together, these three patterns explained 25.5 % of the variance in the dietary intake of study participants, which was comparable to the explained variance identified in previous studies ranging between 15 and 43 % [6, 10]. The food constituents of the Western pattern identified in this study were similar to those typically reported in westernized DPs, such as refined grains, fried potato, fast food, sweets and soda beverages [10, 36–38]; yet the eggs, red and processed meats, as well as alcohol were part of the High Protein/Alcohol pattern, as seen in other studies [39, 40]. The Lebanese pattern, on the other hand, was characterized with a high intake of fruits, vegetables, legumes, nuts and seeds, and bulgur (parboiled whole wheat); food components that have been commonly reported within ‘healthy’ and ‘prudent’ DPs in previous studies conducted in Lebanon, Iran, and the United States respectively [28, 29, 41, 42]. In addition, fruits, vegetables, whole grains, nuts, and seeds have been considered as protective dietary factors associated with lower cardio-metabolic mortality in countries of the Middle East and North Africa (MENA) region [43]. Furthermore, these food groups have been established as key ingredients for healthy aging and longevity through being consistently associated with better cardiometabolic and cognitive health parameters [11].

The present study explored further the characteristics of the three identified DPs in terms of their energy- and nutrient- content as well as diet quality. The Western pattern reflected characteristics of an unhealthy diet as it correlated positively with energy intake and sodium and negatively with the majority of essential micronutrients, including iron, zinc, as well as, lipid- and water-soluble vitamins, except for Vit B12. In addition, this pattern had the lowest correlations with a number of DQIs, including AHEI and aMED, compared to the Lebanese and Protein/Alcohol patterns. In fact, the Western pattern derived in the present study shared comparable food compositions with the ‘fast food/dessert’ and ‘refined grains & desserts’ patterns identified earlier among Lebanese adults (>18 years old), which in turn were similarly associated with high energy consumption, low fiber intake, and irregular breakfast consumption [6, 44]. According to Schroder et al. (2008), the intakes of vitamins and minerals, among older men and women, including folate, calcium, magnesium, iron, zinc, and B vitamins, decrease as the energy density of their diets increase [45]. Furthermore, energy-dense foods and beverages consumed within the westernized DP are known to be of lower nutritional quality prohibiting its consumers, particularly older adults, from meeting their dietary requirements [10, 46].

In contrast to the Western DP, the Lebanese reflected characteristics of a healthy pattern as it had positive correlations with all DQIs considered in the study and had the highest correlations with a number of micronutrients, namely fiber, folate, and vitamin C. Research shows that the Lebanese pattern, comprised mainly of fruits, vegetables, bulgur, dried fruits, legumes, and olive oil, shares common features with the Mediterranean DP [28]; the latter being well-established as a ‘healthy’ pattern that is associated with improved diet quality [47] and reduced risk of diet-related chronic diseases [48–50]. A previous study conducted in Lebanon on rural adults aged 40–60 years showed that the Mediterranean diet was negatively associated with abdominal obesity; however researchers noticed an overall low adherence to the Mediterranean DP among their study participants [51]. These findings were further validated by Belahsen et al. [52], who reported that populations living in Southern Mediterranean countries, including Jordan, Syria and Lebanon, are gradually moving away from their traditional healthy Mediterranean diets and transitioning towards inadequate dietary intakes and more sedentary behaviors [52]. Rapid urbanization and increased availability and access to industrial food are indicated as the major culprits of this nutrition and health transition [28]. In addition to these factors, older adults face a myriad of economic, social, and environmental challenges, such as poverty, food insecurity, and limited access to social safety net programs [4, 53–55], all of which may further limit their ability to consume diets of good quality.

Multiple linear regression results in this study revealed that the strongest correlates of the Lebanese pattern are female gender, higher education, and physical activity level. In addition, higher crowding index, indicative of lower SES, and smoking were found to be negatively associated with the Lebanese pattern. The Western pattern, on the other hand, was found to be associated with male gender, higher crowding index, low education, and other unfavorable lifestyle behaviors. These findings confirm previously reported positive associations between higher education and income with ‘healthy’, ‘prudent’, and Mediterranean DPs [29, 41, 56]. This could be explained by the fact that those with higher education tend to be more health conscious, motivated, and have increased nutrition knowledge to seek and adopt healthy lifestyle behaviors. Furthermore, adults with high SES may have the purchasing power needed to access healthy and nutritious food as well as the means to be engaged in more active lifestyles [29, 41, 56, 57]. According to previous studies conducted on middle-aged and older adults, women tend to adhere more to healthy patterns compared to men [58, 59] and are more likely to adopt better lifestyle behaviors, such as lower consumption of dietary cholesterol and saturated fats, higher consumption of vegetables, and limited sedentary behaviors [29, 42, 60]. Thus, our findings further validate the proposed concept that adherence to a ‘prudent’ diet reflects an overall healthier lifestyle profile that can be protective of adverse health outcomes [59, 61], whereas adults who adhere to unhealthy dietary patterns, such as the Western DP, tend to adopt unfavorable lifestyle behaviors including smoking [41], alcohol consumption [62], and sedentary behaviors [15].

An inverse association between adherence to a Western DP and presence of chronic diseases was observed in this study. Such an association could be an artifact of reverse causation given the cross-sectional nature of the study. In fact, diagnosed elderly may have increased health consciousness due to their condition and would consequently refrain from consuming western-like dietary patterns [63, 64].

In this study, no significant association between identified DPs and obesity were observed (data not shown). Although in previous studies associations were reported between the Western pattern and obesity, most of these studies were conducted among younger adults [29, 47, 65]. The evidence among older adults is less conclusive [10, 58]. In fact, the association between diet and obesity among elderly may be complicated by a myriad of complex biological, hormonal, and metabolic changes. These age-related alterations affect body composition, including the accumulation of body fat and reduction in muscle mass. Furthermore, older adults represent a heterogeneous group with wide individual variations in terms of their physical health, disease progression status, psychosocial conditions, and the use of medications; all of which are factors that may further mask the effect of diet on body weight and obesity [36, 66, 67].

Though, in this study, no association was found between dietary intake and obesity, recent evidence highlight the role of a better quality of diet in preserving and improving physical and cognitive resilience of older adults and promoting healthy aging [68]. In addition to other behavioral factors such as physical activity, diets of high quality may slow down the rate of biological aging at the cellular, molecular, and overall system level and may also reduce the impact of stress on the brain and hence delay age-related vascular cognitive impairments including depression and dementia among older adults [69, 70].

Several strengths exist in this study. It is the first study in Lebanon and the MENA region to identify and characterize the dietary patterns and diet quality of older adults. Data used in this study was derived from a national representative survey with a high response rate. Furthermore, anthropometric and dietary data was collected by trained dietitians resulting in consistent interpretations as well as higher response and completion rates that enhanced the validity of the collected data. However, this study has a number of limitations including possible recall bias and underreporting commonly faced when assessing dietary intake of adults, particularly those who are overweight or obese [71]. In addition, the cross-sectional design of the present study does not allow for inferring causality when exploring associations between identified DPs and various socio-demographic, anthropometric and lifestyle variables. Although the lack of association between the DPs and BMI could be attributed to age-related physiological changes, the high percentage of overweight and obese participants (approximately 80 %) may have limited the variability of the outcome measure (BMI), possibly leading to a null association. Future longitudinal studies are needed to examine changes in DPs over time and to explore diet-disease associations taking into consideration various metabolic biomarkers and body composition indicators. Furthermore, measures of weight change (ex. annual weight change) rather than single and static weight measurements, like BMI, taken at only one point in time may help address the dynamic interplay between diet, obesity, and chronic diseases among older adults [72].

## Conclusion

In conclusion, three main DPs were identified among a representative sample of Lebanese older adults, namely: a Lebanese pattern, a Western and a High Protein/Alcohol pattern. The identified Lebanese pattern, characterized by high intake of fruits, vegetables, legumes, nuts, and seeds, was found to be not only of better dietary quality but it was also associated with healthier lifestyle behaviors and an overall healthy profile compared to the two other DPs. Findings from this study can be the impetus for developing evidence-based public health strategies to promote the Lebanese pattern as a balanced and nutritious diet of high quality in an attempt to promote longevity andoverall quality of life among older adults.

### Ethics approval and consent to participate

This study complies with ethical standards regarding human subject research and was reviewed and approved by the American University of Beirut Institutional Review Board. All participants signed consent forms after being informed of the study objectives and procedures and of their right to withdraw from the study at any time.

### Data availability

Data are available from the authors on request.

## Appendix: Food grouping used in factor analysis

**Table 5:** Food grouping used in factor analysis

Food group	Food items
Alcohol	Non-wine alcoholic beverages, beer, wine.
Bread whole	All types of whole wheat bread.
Breakfast cereals	Regular corn flakes.
Bulgur	Parboiled crushed wheat.
Dairy	Milk, yogurt, strained yogurt (labneh), cheese.
Dried fruits	Raisins, prunes, apricots, dates.
Eggs	All types of cooked eggs.
Fast food	Chawarma sandwiches, falafel sandwiches, hamburger.
Fat	Vegetable oil, butter, mayonnaise.
Fish	All types of cooked fish.
Fried potato	Potato fried, potato chips.
Fruit	Deep yellow orange fruits, banana, apple, strawberry, citrus, grapes, fruit juices fresh.
Fruit juices bottled	All types of sweetened processed fruit drinks.
Hot drinks	Cocoa, Nescafe, tea, turkish coffee.
Legumes	Lentils, beans, fava beans, chickpeas.
Meat	Meat, ‘kebbe’, cured meat.
Nuts and seeds	Salted roasted nuts and seeds.
Olives	Pickled olives.
Pizza and pies	Pizza, manaeesh.
Poultry	All types of cooked chicken.
Refined grains	Bread white, rice and rice products, cooked pasta.
Soda light	All types of soft drinks with sugar substitute.
Soda regular	All types of sweetened soft drinks.
Starchy vegetables	Potato, corn, peas.
Sweets	Arabic sweets, cakes, cookies, doughnuts, ice cream.
Vegetables	Dark green yellow vegetables, tomato, zucchini, eggplant, cauliflower.

## Abbreviations

AHE: alternative healthy eating index
aMED: alternate Mediterranean diet score
DDS: dietary diversity score
DP: dietary patterns
DQI: diet quality index
LMD: Lebanese Mediterranean diet
MENA: Middle East-North Africa
